# Role of Non-coding RNAs on the Radiotherapy Sensitivity and Resistance of Head and Neck Cancer: From Basic Research to Clinical Application

**DOI:** 10.3389/fcell.2020.637435

**Published:** 2021-02-11

**Authors:** Xixia Zhang, Jing Yang

**Affiliations:** Department of Otolaryngology Head and Neck Surgery, Shengjing Hospital of China Medical University, Shenyang, China

**Keywords:** head and neck cancer, radiotherapy, non-coding RNA, miRNA, long non-coding RNA, circRNA

## Abstract

Head and neck cancers (HNCs) rank as the sixth common and the seventh leading cause of cancer-related death worldwide, with an estimated incidence of 600,000 cases and 40–50% mortality rate every year. Radiotherapy is a common local therapeutic modality for HNC mainly through the function of ionizing radiation, with approximately 60% of patients treated with radiotherapy or chemoradiotherapy. Although radiotherapy is more advanced and widely used in clinical practice, the 5-year overall survival rates of locally advanced HNCs are still less than 40%. HNC cell resistance to radiotherapy remains one of the major challenges to improve the overall survival in HNC patients. Non-coding RNAs (ncRNAs) are newly discovered functional small RNA molecules that are different from messenger RNAs, which can be translated into a protein. Many previous studies have reported the dysregulation and function of ncRNAs in HNC. Importantly, researchers reported that several ncRNAs were also dysregulated in radiotherapy-sensitive or radiotherapy-resistant HNC tissues compared with the normal cancer tissues. They found that ectopically elevating or knocking down expression of some ncRNAs could significantly influence the response of HNC cancer cells to radiotherapy, indicating that ncRNAs could regulate the sensitivity of cancer cells to radiotherapy. The implying mechanism for ncRNAs in regulating radiotherapy sensitivity may be due to its roles on affecting DNA damage sensation, inducing cell cycle arrest, regulating DNA damage repair, modulating cell apoptosis, etc. Additionally, clinical studies reported that *in situ* ncRNA expression in HNC tissues may predict the response of radiotherapy, and circulating ncRNA from body liquid serves as minimally invasive therapy-responsive and prognostic biomarkers in HNC. In this review, we aimed to summarize the current function and mechanism of ncRNAs in regulating the sensitivity of HNC cancer cells to radiotherapy and comprehensively described the state of the art on the role of ncRNAs in the prognosis prediction, therapy monitoring, and prediction of response to radiotherapy in HNC.

## Introduction

Head and neck cancers (HNCs) rank as the sixth common and the seventh leading cause of cancer-related death worldwide, with an estimated incidence of 600,000 cases and 40–50% mortality rate every year (Ferlay et al., 2015; Economopoulou et al., 2016). HNCs are presented mainly by the squamous cell carcinoma originating from the epithelial cells of the oral cavity, nasal cavity, oropharynx, larynx, or hypopharynx (Leemans et al., 2018).

Radiotherapy is a common local therapeutic modality for cancers mainly through the function of ionizing radiation. Ionizing radiation could kill cancer cells via directly damaging the DNA strands of cancer cells to end the infinite proliferative capacity, and also indirectly causes DNA damages in cancer cell through ionizing water to generating highly reactive oxygen species. Radiotherapy is one of the major treatment modalities for HNC, with approximately 60% of patients treated with radiotherapy or chemoradiotherapy (Berrington de Gonzalez et al., 2011). Many high-quality clinical studies have reported that radiotherapy or concurrent chemoradiotherapy could improve the survival for patients with early-stage HNC or locally advanced HNC (Bonner et al., 2010; Caudell et al., 2017; Lacas et al., 2017; Nichols et al., 2019). Although radiotherapy is more advanced and widely used in clinical practice, 5-year overall survival rates of locally advanced HNC are still less than 40% (Pignon et al., 2009). Cancer cell resistance to radiotherapy remains one of the major challenges to improve the overall survival in HNC patients.

Non-coding RNAs (ncRNAs) are newly discovered functional RNA molecules that are different from messenger RNAs, which can be translated into a protein (Mattick and Makunin, 2006; Feng et al., 2019; Dai et al., 2020; Yu et al., 2020). Over the past decades, researchers have discovered multiple kinds of ncRNAs, showing that ncRNAs constitute more than 90% of RNAs transcribed from the human genome DNA (Slack, 2006). Such discovery of numerous ncRNAs has opened up brand-new directions for understanding the normal physiology and the development of diseases. Based on the function difference, ncRNAs are usually divided into two categories: housekeeping and regulatory ncRNAs. The regulatory ncRNAs are mainly composed of miRNAs, circRNAs, and long non-coding RNAs (lncRNA; Chen, 2016; Wu et al., 2017; Yao et al., 2019). In recent years, regulatory RNAs were extensively researched and are revealed to participate in regulating the expression of protein-coding genes at transcriptional, post-transcriptional, as well as translational levels. Emerging studies have reported the key roles of regulatory ncRNAs in various biological processes, disease occurrence, and development.

Many previous studies have reported the dysregulation and function of ncRNAs in HNC (Wang et al., 2018; Jiang et al., 2019; Vo et al., 2019). NcRNAs could regulate the expression of genes associated with cell cycle, cell apoptosis, invasion, and migration, and eventually affect the proliferation, invasion, and metastasis of HNC (Feng et al., 2020; Gu et al., 2020; Huang et al., 2020; Sur et al., 2020; Wang et al., 2020; Zhang et al., 2020). Additionally, researchers reported that several ncRNAs were also dysregulated in radiotherapy-sensitive or radiotherapy-resistant HNC tissues compared with the normal cancer tissues (Zhang et al., 2013; de Jong et al., 2015; Qu et al., 2015a, b; Suh et al., 2015; Xu et al., 2015; Gao et al., 2017a; Hess et al., 2017; Chen et al., 2019; Vahabi et al., 2019; Pasi et al., 2020). They found that ectopically elevating or knocking down expression of some ncRNA could significantly influence the response of HNC cancer cells to radiotherapy, indicating that ncRNAs could regulate the sensitivity of cancer cells to radiotherapy. In this review, we aimed to summarize the current function and mechanism of ncRNAs in regulating the sensitivity of HNC cancer cells to radiotherapy.

## Radiation-Induced Cell Response and Mechanism of Radiotherapy Resistance

Since the discovery of ionizing radiation in 1895, the concept of radiation-based therapy modality was prompted and has been regarded as a major treatment for many types of cancers (Wills, 1904; Alsahafi et al., 2020; Ampil et al., 2020; Plavc et al., 2020; Tao et al., 2020; Sher et al., 2020a, b). Especially, radiotherapy is a crucial treatment for HNC. The radiation-induced cell death is regarded to originate from the damage of two cellular components: DNA and cell membrane.

Upon exposure to radiation, the radiation passed directly through the cell and ionized the DNA, causing lethal DNA damage. Additionally, the radiation can also result in DNA damage through ionizing the intracellular water and inducing the generation of reactive oxygen species (Rothkamm and Löbrich, 2003). These reactive oxygen species could bring various injuries to cells, among which are the DNA double-strand breaks (DSBs), which are one of the most cytotoxic injuries to cells. DSBs, involving breaks in the phosphodiester backbone of both strands of DNA, increase positively with the radiation dose (Hanai et al., 1998; Rothkamm and Löbrich, 2003). It is estimated that each gray unit of radiation could produce 10^5^ ionizations per cell, which cause about 40 DSBs, 2,000 single-strand breaks (SSBs), as well as other types of damages in the DNA (Lewanski and Gullick, 2001). When recognizing these complex radiation-induced damages, the cells respond to these damages through multiple signaling pathways. These pathways are involved in modulating important cellular activities such as DNA damage repair, cell-cycle arrest, and apoptosis. In addition, radiation could also function on the cell membrane to mediate cell apoptosis. Mechanistically, radiation activates the enzyme sphingomyelinase, which could hydrolyze plasma-derived sphingomyelin and produce ceramide. Through this process, ceramide could be produced within seconds of radiation exposure. The accumulation of ceramide in cells could initiate cell apoptosis (Obeid et al., 1993; Haimovitz-Friedman et al., 1994; Jarvis et al., 1994a, b). However, the mechanism on how ceramide initiates apoptosis is still unclear.

In response to radiation exposure, most cells immediately initiate cell apoptosis because of severe DNA damage or the accumulation of ceramide in cells. Such programmed death mechanism could protect cells from propagating genetic mutations to the next generation. Additionally, cells will also initiate the DNA damage repair system, which was developed during the evolution of species. There are two major DNA repair pathways: homologous recombination (HR) and non-homologous end joining (NHEJ; Burgess et al., 2020; Nastasi et al., 2020; Zhao et al., 2020).

The HR repairing system depends on homologous DNA sequences from sister chromatids. This makes HR restricted to phases of the cell cycle where homologous sister chromatids coexist (Zhao et al., 2017). In contrast, NHEJ is a promiscuous repair system that directly ligates two broken ends independent of sequence homology. Hence, NHEJ is not cell cycle dependent and could be initiated at any cell cycle phase (Emerson and Bertuch, 2016). Another important response to DNA damage is cell cycle arrest. Normally, cell cycles pass through the G0 phase, G1 phase, S phase, G2 phase, and M phase. The cell cycle checkpoint pathway regulated the progression of the cell cycle. When sensing DNA damages, cells will stop the cell cycle progression to spare more time for DNA repairing. Overall, these complex cell responses to radiation-induced injury depend on the modulation of various genes.

The sensitivity of cancer cells to radiotherapy depends on the intensity of DNA damage with cells, the cells’ ability to balance the expression of genes associated with apoptosis promotion and inhibition, the expression level of genes regulating the induction of cell cycle arrest, and the DNA repair system. Radiotherapy-resistant cancer cells showed obvious tendency to inhibit cell apoptosis and augment the DNA damage repair rate. This adaptation to radiotherapy is closely related with the dysregulated expression of genes in resistant cells. NcRNAs, especially the regulatory ncRNAs, are characterized for their role on regulating gene expression in cancer cells. Because of such an important role, ncRNAs are believed to be associated with radiotherapy resistance and could regulate cell sensitivity to radiotherapy.

## Ionizing Radiation Modulates Ncrna Expression Profiling

The role of ncRNAs on cancer development and progression is one of the hottest topics in cancer cell biology research. With the help of microarray and next-generation technology, researchers have discovered an amount of ncRNAs, such as miRNAs, lncRNAs, and circRNAs, which was differently expressed in cancer tissues compared with the normal tissues. Many of these ncRNAs could significantly regulate the biological behaviors of cancer cell. In HNC, many researchers also focus on the expression and function of ncRNAs. Numerous ncRNAs are reported to modulate HNC proliferation, invasion, metastasis, apoptosis, and other biological processes (Wu et al., 2015; Bao et al., 2018; Vo et al., 2019; Wang S. S. et al., 2019). Recently, researchers also found that these oncogenic or tumor-suppressive ncRNAs could also regulate the sensitivity of cancer cells to radiotherapy. Moreover, they also explore dysregulated ncRNAs expression through comparing radiotherapy-resistant, radiotherapy-sensitive, and normal cancer tissues or cells, so as to discover key ncRNAs that could regulate radiotherapy sensitivity. We summarized the differently expressed ncRNAs in radiotherapy-resistant, radiotherapy-sensitive, and normal cancer tissues or cells as shown in Table 1 (Qu et al., 2012, 2015a,b; Li et al., 2013; Li B. Y. et al., 2017; Li L. N. et al., 2017; Li et al., 2020; Shiiba et al., 2013; Zhang et al., 2013; Wang et al., 2014, 2016, Wang Z. et al., 2019; de Jong et al., 2015; Lin et al., 2015; Maia et al., 2015; Suh et al., 2015; Sun et al., 2015; Xu et al., 2015; Zhao et al., 2015; Huang et al., 2016, 2018, 2019; Kang et al., 2016; Weng et al., 2016; Wu et al., 2016, 2018; Gao et al., 2017a, b; Han et al., 2017; Hu et al., 2017; Chen H. et al., 2018; Chen et al., 2019; Feng et al., 2018; He et al., 2018; Yang et al., 2018; Kong et al., 2019; Tian et al., 2019; Vahabi et al., 2019; Yi et al., 2019; Gou et al., 2020; Kangboonruang et al., 2020; Shuai and Huang, 2020).

**TABLE 1:** Dysregulated ncRNAs in response to radiation in HNC.

Studies	ncRNA	Expression of ncRNA in response to radiation	Cancer type
Qu et al., 2012	miR-205	Up-regulation	NPC
Li et al., 2013	miR-324-3p	Down-regulation	NPC
Zhang et al., 2013	miR-29c	Down-regulation	NPC
Wang et al., 2014	miR-24	Down-regulation	NPC
Lin et al., 2015	miR-378g	Down-regulation	NPC
Qu et al., 2015a	miR-23a	Down-regulation	NPC
Qu et al., 2015b	miR-203	Down-regulation	NPC
Sun et al., 2015	miR-101	Down-regulation	NPC
Xu et al., 2015	miR-185-3p, miR-324-3p	Down-regulation	NPC
Zhao et al., 2015	miR-504	Up-regulation	NPC
Huang et al., 2016	miR-19b-3p	Up-regulation	NPC
Kang et al., 2016	miR-24	Up-regulation	NPC
Wang et al., 2016	miR-24-3p	Up-regulation	NPC
Gao et al., 2017a	miR-138-5p	Down-regulation	NPC
Gao et al., 2017b	ebv-miR-BART7	Up-regulation	NPC
Han et al., 2017	XIST	Up-regulation	NPC
Hu et al., 2017	lncRNA ANRIL	Up-regulation	NPC
Li B. Y. et al., 2017	miR-210	Up-regulation	NPC
Li L. N. et al., 2017	miR-125b	Up-regulation	NPC
Feng et al., 2018	miR-495	Down-regulation	NPC
He et al., 2018	lnRNA PVT1	Up-regulation	NPC
Huang et al., 2018	miR-150	Up-regulation	NPC
Wu et al., 2018	miR-222	Up-regulation	NPC
Chen et al., 2019	CircRNA_000543	Up-regulation	NPC
Kong et al., 2019	miR-193a-3p	Up-regulation	NPC
Tian et al., 2019	miR-483-5p	Up-regulation	NPC
Wang et al., 2018	miR-372	Down-regulation	NPC
Yi et al., 2019	lncRNA PTPRG-AS1	Up-regulation	NPC
Shuai and Huang, 2019	circRNA_001387	Up-regulation	NPC
Maia et al., 2015	miR-296-5p	Up-regulation	Laryngeal cancer
Chen H. et al., 2018	miR-128a	Down-regulation	Laryngeal cancer
Yang et al., 2018	lncRNA-NKILA	Down-regulation	Laryngeal cancer
Shiiba et al., 2013	miR-125b	Down-regulation	Oral cancer
Weng et al., 2016	miR-494-3p	Down-regulation	Oral cancer
Wu et al., 2016	miR-17-5p	Up-regulation	Oral cancer
de Jong et al., 2015	microRNA Expression	Down-regulation	HNC
Suh et al., 2015	miR-196a	Up-regulation	HNC
Huang et al., 2019	LINC00958	Up-regulation	HNC
Vahabi et al., 2019	miR-96-5p	Up-regulation	HNC
Gou et al., 2020	lncRNA BLACAT1	Up-regulation	HNC
Kangboonruang et al., 2020	MALAT1	Up-regulation	HNC
Li et al., 2020	LINC00520	Up-regulation	HNC

*HNC, head and neck cancer; NPC, nasopharyngeal carcinoma.*

## ncRNAs Directly Regulate Radiotherapy Sensitivity by Modulating Specific Processes

The efficacy of radiotherapy depends mainly on the sensitivity of cancers to radiotherapy. Many factors are found to influence the radiotherapy sensitivity of cancers. Several research groups have discovered that some differentially expressed ncRNAs in HNC could affect radiotherapy sensitivity. Meanwhile, several research groups also discovered differentially expressed ncRNAs in radiation-sensitive and radiation-resistant HNC cancer cells by sequencing and microarray analysis. Some high-regulated ncRNAs in radiation-resistant cancers could decrease the sensitivity of cancer cells to radiotherapy, whereas ncRNAs down-regulated in radiation-resistant cancers could enhance the sensitivity of cancer cells to radiotherapy. These ncRNAs modulate the response of cancer cells to radiotherapy mainly through regulating different genes involved in important processes that are tightly associated with radiotherapy sensitivity. In this section, we mainly describe the effect and molecular mechanisms of ncRNA on regulating radiotherapy sensitivity of HNC cells by DNA damage sensing, cell cycle progression, DNA damage repair, and cell apoptosis of HNC cells.

### Affecting DNA Damage Sensation and Inducing Cell Cycle Arrest

**FIGURE 1:**
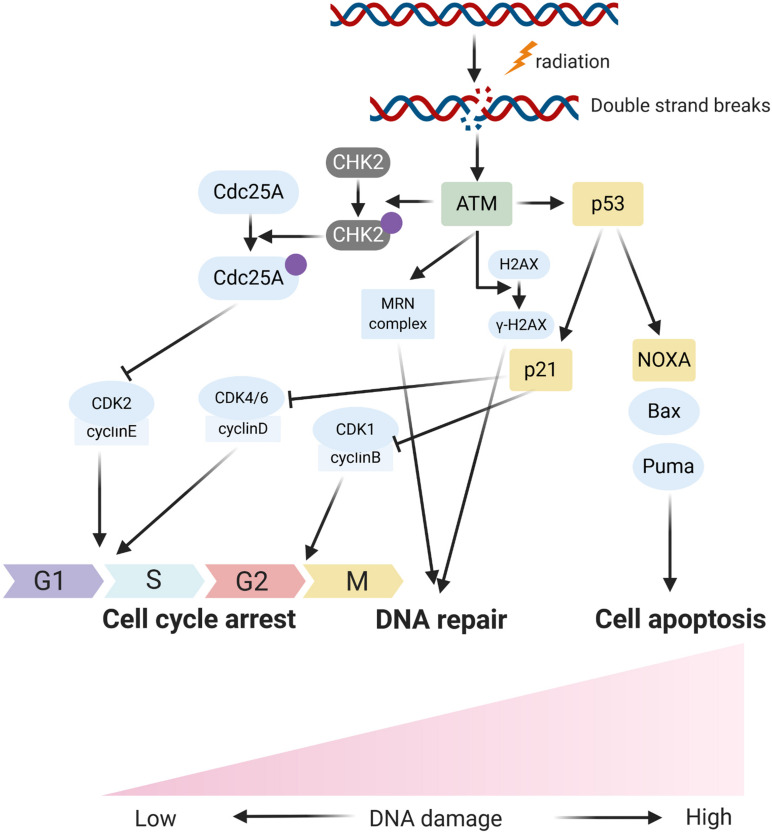
The schematic diagram fors main respones of cells to radiation induced DNA damage.

**FIGURE 2:**
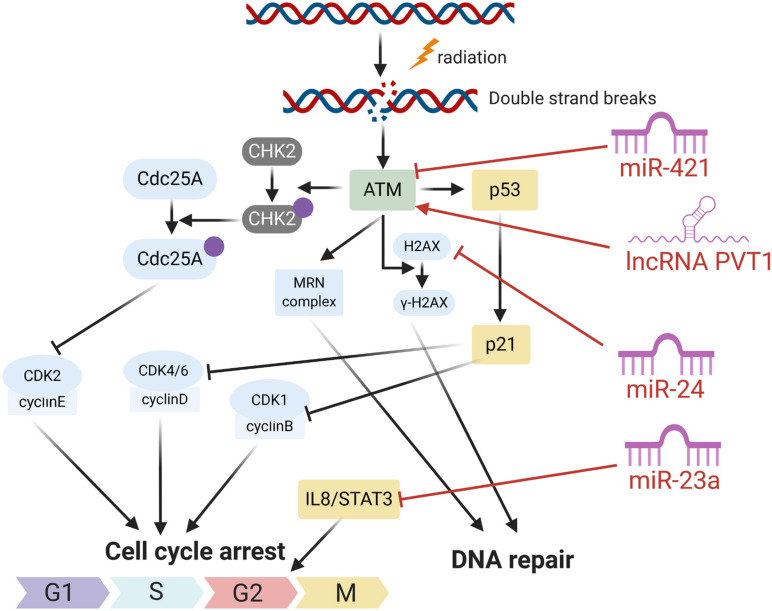
The roles of several ncRNAs on cell respones radiation induced DNA damage.

In response to DNA damage, cells have evolutionarily developed a mechanism to sense the DNA damage and initiate DNA damage repair. In the process, the ataxia telangiectasia mutated (ATM), an important Ser/Thr kinase, plays a major role in sensing DNA DSBs and initiating a series of cascade responses leading to cell cycle checkpoint activation and DNA repair (Weber and Ryan, 2015; Carrassa and Damia, 2017; Figures 1, 2). After sensing the radiation-induced DNA damage, the main sensor ATM and the ataxia telangiectasia mutated and Rad3 related (ATR) were activated and subsequently activated the cell cycle checkpoint kinases. Usually, ATM activates the checkpoint kinase 2 (CHK2), and ATR is responsible for the activation of CHK1. The activation of CHK1/2 could lead to phosphorylation and inactivation of CDC25A and CDC25C, both of which were involved in dephosphorylation and activation of CDK2 and CDK1, respectively. Inactivation of CDC25A and CDC25C consequently leads to maintenance of CDKs in the phosphorylated and inactivated form, thus inhibiting S phase and M phase entry and eventually inducing cell cycle arrest. Overall, ATM/CHK2 and ATR/CHK1 pathways work coordinately and cooperate to mediating cellular responses to DNA damage and are responsible for the maintenance of genomic stability by inducing cell cycle arrest. In HNC, the function of ATM was reported to be directly and indirectly regulated by several ncRNAs including miRNA and lncRNA. Mansour et al. (2013) reported that miR-421 could directly inhibit the expression of ATM in HNC through binding the ATM’ S 3’-untranslated region (UTR). Forced expression of this miRNA could result in a significant cellular ATM deficiency and defect DNA damage repair, and eventually promote the sensitivity of HNC cells to radiotherapy. LncRNA PVT1 was found to be overexpressed in HNC tissues and cell line, and knockdown of PVT1 enhances the radiosensitivity of nasopharyngeal carcinoma (NPC) cell lines (He et al., 2018). Mechanically, knockdown of PVT1 significantly decreased the phosphorylation levels of ATM, p53, and Chk2, causing a decrease in the DNA repair ability of NPC cells after radiotherapy and enhancing their radiosensitivity (He et al., 2018).

### Regulating DNA Damage Repair

When DSBs, the most harmful type of DNA damage, occurred in cells, the histone variant H2AX was immediately phosphorylated by the activated ATM (Jackson, 2002; Symington and Gautier, 2011). Therefore, phosphorylated H2AX (γ-H2AX) can be a useful indicator for DSB DNA damage. More importantly, γ-H2AX is responsible for the recruitment of DNA repair associated proteins to initiate the NHEJ or HR repair processes. In NPC cancer, miR-24 has been revealed to directly target H2AX (Wang et al., 2014). It demonstrated that the inhibition of miR-24 results in significant up-regulation of H2AX and thereby renders cancer cells resistant to radiation-induced DNA damage (Wang et al., 2014). Meanwhile, researchers also reported that miR-24 can inhibit the DNA DSB repair by targeting a DNA damage repair associated protein c-Jun activation domain binding protein-1 (Jab1; Wang et al., 2016). Jab 1 was crucial for DSB repair, and deletion of Jab1 resulted in spontaneous DNA fragmentation and increased expression of the γ-H2AX level (Doronkin et al., 2002; Pan et al., 2012, 2013).

### Modulation of Cell Apoptosis

When the radiation-induced DNA damage is too lethal to be repaired, cells would initiate an automatic death program like apoptosis to protect genome stability. Therefore, the flexibility of cancer cells to initiate cell apoptosis sometimes may determine whether cancer cells are sensitive to radiotherapy or resistant to it. Some ncRNAs have been reported to influence the sensitivity of cancer cells to radiotherapy through modulation of cell apoptosis. Mechanistically, these ncRNAs mainly function through regulating the expression of anti-apoptosis or pro-apoptosis members (Table 2).

**TABLE 2:** ncRNAs regulating the expression of anti-apoptosis or pro-apoptosis members.

Studies	ncRNA	Cancer type	Target genes	Effects on apoptosis (overexpression)	Effects on radiotherapy (overexpression)
Qu et al., 2012	miR-205	NPC	PTEN	Inhibiting cell apoptosis	Radiotherapy resistance
Zhang et al., 2013	miR-29c	NPC	mcl1 or bcl2	Pro-apoptosis	Radiotherapy sensitive
Wang et al., 2014	miR-24	NPC	H2AX	Pro-apoptosis	Radiotherapy sensitive
Lin et al., 2015	miR-378g	NPC	SHP-1	Pro-apoptosis	Radiotherapy sensitive
Xu et al., 2015	miR-185-3p, miR-324-3p	NPC	SMAD7	Pro-apoptosis	Radiotherapy sensitive
Zhao et al., 2015	miR-504	NPC	NRF1	Inhibiting cell apoptosis	Radiotherapy resistance
Huang et al., 2016	miR-19b-3p	NPC	TNFAIP3/NF-κB axis	Inhibiting cell apoptosis	Radiotherapy resistance
Hu et al., 2017	lncRNA ANRIL	NPC	miR-125a	Inhibiting cell apoptosis	Radiotherapy resistance
Li B. Y. et al., 2017	miR-210	NPC	HIF-1α, CTR1A, ADAMTS5, CAMTA1	Inhibiting cell apoptosis	Radiotherapy resistance
He et al., 2018	lnRNA PVT1	NPC	ATM-P53	Inhibiting cell apoptosis	Radiotherapy resistance
Kong et al., 2019	miR-193a-3p	NPC	SRSF2	Inhibiting cell apoptosis	Radiotherapy resistance
Yi et al., 2019	lncRNA PTPRG-AS1	NPC	miR-194-3p/PRC1	Inhibiting cell apoptosis	Radiotherapy resistance
Yang et al., 2018	lncRNA-NKILA	Laryngeal cancer	NFKB/ikb/p65 Pathway	Pro-apoptosis	Radiotherapy sensitive
Wu et al., 2016	miR-17-5p	Oral cancer	p53	Inhibiting cell apoptosis, G2/M phase arrest	Radiotherapy resistance
Gou et al., 2020	lncRNA BLACAT1	HNC	PSEN	Inhibiting cell apoptosis, G2/M phase arrest	Radiotherapy resistance
Li et al., 2020	LINC00520	HNC	LINC00520/miR-195/HOXA10	Inhibiting cell apoptosis, G2/M phase arrest	Radiotherapy resistance

*HNC, head and neck cancer; NPC, nasopharyngeal carcinoma.*

**FIGURE 3:**
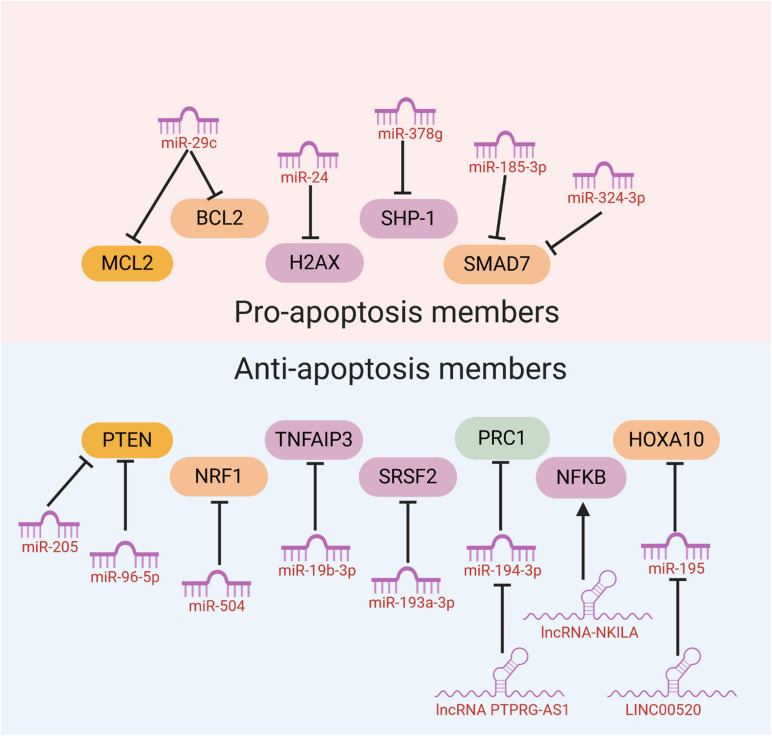
NcRNAs could affect HNC cancer cell sensitivity to radiotherapy through modulation of cell apoptosis.

Many ncRNAs could enhance HNC cancer cell sensitivity to radiotherapy through promoting cell apoptosis. MiR-29c was found to target the classical anti-apoptotic proteins B cell lymphoma 2 (Bcl-2) family including myeloid cell leukemia 1 (MCL1) and Bcl-2 itself in human NPC. *In vitro* and *in vivo* studies illustrated that miR-29c could promote cell apoptosis, through which ectopic restoration of miR-29C substantially enhanced the sensitivity of NPC cells to radiotherapy (Zhang et al., 2013). MiR-378g was reported to enhance radiosensitivity, promoting apoptosis in NPC cells via directly targeting SHP-1 (Lin et al., 2015). Overexpression of SHP-1 partially reversed the effect of miR-378g mimics on cell apoptosis and radiosensitivity. MiR-185-3p and miR-324-3p can inhibit NPC cell growth and promote apoptosis partly through targeting SMAD7 (Xu et al., 2015; Figure 3).

On the other hand, several miRNAs were reported to inhibit cell apoptosis and enhance HNC cancer cell resistance to radiotherapy. Evidence has shown that PTEN is involved in regulating cell response to radiation-induced cell apoptosis and cell cycle arrest through inhibiting radiation-induced activation of the PI3K-Akt signaling pathway. Several miRNAs including miR-205 and miR-96-5p were reported to be elevated in HNC tissues from NPC patients after radiotherapy, and target PTEN to mediate resistance of HNC cells to radiotherapy (Qu et al., 2012; Vahabi et al., 2019). MiR-504 is found to be up-regulated in NPC radioresistant cells and could directly inhibit the expression of NRF1 and lead to radioresistance in NPC cells. NRF1 inhibition by miR-504 disturbed mitochondrial-mediated oxidative responses, which influence apoptosis of HNC cells and contribute to the resistance of cancer cells to radiation (Zhao et al., 2015). Huang et al. revealed that miR-19b-3p is up-regulated in NPC and could activate NF-κB activity by targeting TNFAIP3. This miR-19b-3p/TNFAIP3/NF-κB axis could eventually inhibit cancer cell apoptosis and lead to NPC cell radioresistance (Huang et al., 2016). In addition, miR-210, miR-193a-3p, and miR-17-5p were reported to inhibit HNC cell apoptosis and promote cancer cell resistance to radiotherapy (Wu et al., 2016; Li B. Y. et al., 2017; Kong et al., 2019; Figure 3).

Recently, evidences have discovered that several lncRNAs and circRNAs could influence cancer cell sensitivity to radiotherapy through modulation of cell apoptosis. These lncRNAs and circRNAs function mainly through alleviating the role of certain miRNAs, which could be bound by lncRNAs and circRNAs through the base-pairing principles. For example, lncRNA ANRIL could function as a miR-125a sponge and negatively modulate miR-125a expression. ANRIL could reverse the inhibited proliferation, induced apoptosis, and enhanced radiosensitivity triggered by miR-125a overexpression (Hu et al., 2017). PTPRG-AS1 was found to specifically bind to miR-194-3p as a competing endogenous RNA, and miR-194-3p negatively regulates PRC1. Silencing PTPRG-AS1 could release the expression of miR-194-3p and resulted in enhanced sensitivity to radiotherapy and cell apoptosis (Yi et al., 2019). The LINC00520 could promote cell resistance to radiotherapy through reversing miR-195/HOXA10 in HNC (Li et al., 2020). CircRNA_000543 could serve as a sponge for miR-9 in NPC. Silencing circRNA_000543 sensitizes NPC cells to radiation by targeting the miR-9/PDGFRB axis (Chen et al., 2019). Additionally, NF-κB interacting lncRNA (NKILA) has been reported to be down-regulated in laryngeal cancer and could enhance the cytotoxicity of radiation through promoting cell apoptosis. Mechanically, lncRNA NKILA functions through combining with NF-κB: IκB complex to inhibit IκB phosphorylation, inhibits p65 nuclear translocation, and finally inhibits NF-κB activation (Yang et al., 2018; Figure 3).

### Activation of EGFR Signaling

Ionizing radiation activates the epidermal growth factor receptor (EGFR) family of receptor tyrosine kinases, which, in turn, can initiate PI3K/AKT or MAPK pathways.

**FIGURE 4:**
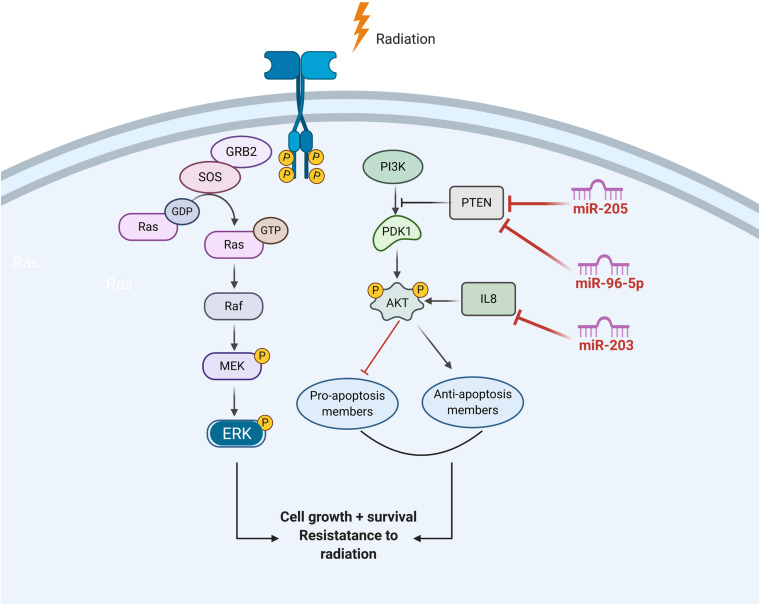
The role of ncRNAs on the regualtion of EGFR signalling in response to radiation induced DNA damage.

Through activating the PI3K-AKT pathway, EGFR signaling can prevent radiation-induced apoptosis. EGFR signaling can also promote cancer cell growth by inducing cell cycle progression, which was driven by activation of the retrovirus associated DNA sequence (RAS)–rapidly accelerated fibrosarcoma (RAF)–mitogen/extracellular signal-regulated kinase (MEK)–ERK pathways. Published data demonstrate that miR-203 is a critical determinant of NPC cells’ response to radiotherapy, and reduced miR-203 could promote NPC cell radioresistance by activating IL8/AKT signaling (Qu et al., 2015b). In addition, PTEN is a common inhibitor of AKT and is also a direct target of several miRNAs in HNC, such as miR-205 and miR-96-5p (Figure 4).

### Epithelial-to-Mesenchymal Transition

Epithelial-to-mesenchymal transition (EMT) is a phenotypic change in which the epithelial cancer cell acquired a fibroblastoid-like morphology. Such transition could result in enhanced tumor cell motility and invasiveness, increased metastatic potential, as well as resistance to radiotherapy or chemotherapy (Marie-Egyptienne et al., 2013). ZEB1, an important EMT marker, could also be regulated by lncRNA NEAT1 in NPC. lncRNA NEAT1 knockdown could sensitize NPC cells to radiation through releasing the expression of miR-204, and eventually enhances the expression of ZEB1, a downstream target of miR-204.

## Clinical Application of ncRNAs as Biomarkers to Radiotherapy

### The *in situ* ncRNA Expression in HNC Tissues Predicting Response of Radiotherapy

It is well known that ncRNAs have shown an important regulatory role on the sensitivity or resistance of HNC cells to radiotherapy. Based on this, ncRNAs expression can be useful biomarkers to identify HNC patients who will be sensitive to radiotherapy and to predict the survival outcomes of HNC patients receiving radiotherapy in clinical practice. One study explored the associations between miR-200b and miR-155 expression in HNC tissues and outcome, and confirmed the prognosis predictive value of candidate miRNAs (Hess et al., 2017). Additionally, some researchers began to build a predictive panel consisting of multiple markers in an attempt to better improve the predictive value. Chen L. et al. (2018) analyzed a large scale of miRNA array profiles and the corresponding clinical records for HNSCC patients (including 509 carcinomas and 44 normal mucosa specimens) from the TCGA. They established a 5-miRNA signature including miR-99a, miR-31, miR-410, miR-424, and miR-495. Their results showed that this 5-miRNA signature could predict clinical outcomes, and the 5-miRNA signature-based nomogram is useful in predicting radiotherapy response and survival in HNSCC, implying that it might become a promising tool to optimize radiation strategies.

### Circulating ncRNAs Serve as Minimally Invasive Therapy-Responsive and Prognostic Biomarkers

Circulating biomarkers in the peripheral blood, such as the biomarker HSP70 in HNSCC patients (Gehrmann et al., 2014), could provide a minimally invasive way to predict therapy response and survival outcomes, as well as monitor the therapy. NcRNAs, especially miRNAs, were reported to show high stability in blood plasma and resistance to RNase activity (Mitchell et al., 2008). This characteristic of miRNA combined with the minimally invasive accessibility of blood sample makes circulating miRNAs useful and attractive biomarkers. Nakashima et al. (2019) reported that the expression of miR-1290 was significantly down-regulated in the plasma of oral squamous cell carcinoma patients as compared to that in healthy volunteers. The patients who showed a poor pathological response to chemoradiotherapy presented a high proportion of miR-1290 down-regulation (Nakashima et al., 2019), suggesting that miR-1290 expression may be useful for guiding treatment decisions in oral squamous cell carcinoma patients receiving radiotherapy. Another research identified eight plasma miRNAs that differentiated significantly between HNC patients and the healthy donors. These candidate miRNAs also showed well therapy-response features and significantly decreased after receiving radiotherapy.

## Conclusion and Prospective

Radiotherapy is a common therapeutic modality for HNC. Decreased sensitivity or resistance to radiotherapy is still a significant challenge in clinical practice and a barrier to improve the prognosis of HNC patients. With the development of molecular biology and sequencing technology, mounting evidence revealed the important role of ncRNAs on the carcinogenesis, development, and therapy resistance of HNC. These findings were of great significance for the following reasons. First, they highlight the potential of intervening relevant ncRNAs to overcome resistance and re-sensitize cancer cells to the effects of radiotherapy. Second, the ncRNAs could be used as circulating biomarkers in the peripheral blood to predict therapy response and survival outcomes, as well as monitor the therapy.

In the future, more studies are required to further elucidate the potential roles of novel ncRNAs like circRNAs and piRNAs in regulating the sensitivity of HNC cells to radiotherapy. Furthermore, it is still unclear and less studied whether the tumor cells or tumor mesenchymal cells-derived exosomal ncRNAs confer HNC cell resistance characteristics to radiotherapy. These topics will be very interesting and meaningful because circRNAs were more stable than linear RNAs due to their covalently closed loop structures, and exosome encapsulated ncRNAs were less easy to be degraded in the circulation system. These characteristics make circRNAs and exosomal ncRNAs potential biomarker candidates for prognosis prediction and therapy response monitoring. Additionally, with the development of bioinformatics, the integration of ncRNA signature profiling into HNC screening algorithms may help in increasing the specificity of screening patients who will benefit from radiotherapy and improving the prognosis of HNC patients.

## Author Contributions

JY designed the review. XZ and JY collected the related manuscript. XZ and JY drafted and revised the manuscript. Both authors read and approved the final manuscript.

## Conflict of Interest

The authors declare that the research was conducted in the absence of any commercial or financial relationships that could be construed as a potential conflict of interest.

## Abbreviations

ATM: ataxia telangiectasia mutated
ATR: the ataxia telangiectasia mutated and Rad3 related
Bcl-2: B cell lymphoma 2
CHK2: checkpoint kinase 2
DSBs: DNA double-strand breaks
EGFR: epidermal growth factor receptor
EMT: epithelial-to-mesenchymal transition
γ-H 2AX: phosphorylated H2AX
HNCs: head and neck cancers
HR: homologous recombination
Jab1: c-Jun activation domain binding protein-1
lncRNA: long non-coding RNAs
MCL1: myeloid cell leukemia 1
ncRNAs: non-coding RNAs
NHEJ: non-homologous end joining
NKILA: NF- κ B interacting lncRNA
NPC: nasopharyngeal carcinoma
SSBs: single-strand breaks
UTR: untranslated region.
