# Supplementary material for: Role of Non-coding RNAs on the Radiotherapy Sensitivity and Resistance of Head and Neck Cancer: From Basic Research to Clinical Application
Source: Front Cell Dev Biol. 2021 Feb 11;8:637435. doi: 10.3389/fcell.2020.637435 (PMC7905100; doi:10.3389/fcell.2020.637435)
Supplement: Supplementary file 1 [file Data_Sheet_1.PDF]

Supplementary Table 1 circulating ncRNAs markers in HNC

Studies	expression	in situ or circulating	function	Cancer
miR-186-5p	Upregulation (before treatment)	circulating	prognosis, therpy monitoring	HNC
miR-142-3p	Upregulation (after treatment)	circulating	prognosis, therpy monitoring	HNC
miR-195-5p	Upregulation (after treatment)	circulating	prognosis, therpy monitoring	HNC
miR-374b-5p	Upregulation (before treatment)	circulating	prognosis, therpy monitoring	HNC
miR-574-3p	Upregulation (before treatment), Upregulation (after treatment)	circulating	prognosis, therpy monitoring	HNC
miR-28-3p	Upregulation (after treatment)	circulating	prognosis, therpy monitoring	HNC
miR-191-5p	Upregulation (after treatment)	circulating	prognosis, therpy monitoring	HNC
miR-425-5p	Upregulation (after treatment)	circulating	prognosis, therpy monitoring	HNC
miR-1290	Downregulation	circulating	Prognosis, response to therpy	HNC

Notes: HNC, head and neck carcinoma.
